# Determinants of COVID-19 prevalence in Central Java, Indonesia: An ecological study of socio-demographic, environmental, and healthcare factors

**DOI:** 10.1016/j.dialog.2025.100263

**Published:** 2025-12-12

**Authors:** Iqbal Ardiansyah, Agus Subagiyo, Arif widyanto, Army Mitasari

**Affiliations:** a Department of Environmental Health, Poltekkes Kemenkes Semarang, Banyumas, Central Java, 53151, Indonesia; b Environmental Health Student Poltekkes Kemenkes Semarang, Banyumas, Central Java, Indonesia

**Keywords:** Pandemic, Coronavirus disease 2019, Geospatial analysis, Risk factor, Localized

## Abstract

Central Java, Indonesia, experienced a 40.9 % COVID-19 positivity rate in 2022, exceeding the WHO benchmark. This study examines the association between changes in sociodemographic, environmental, and healthcare factors and the rise in COVID-19 prevalence, focusing on regional disparities across Central Java. Variables from public datasets were chosen based on the Social Determinants of Health (SDOH) framework. Data analysis begins with variable identification via Pearson correlation, followed by an Ordinary Least Squares (OLS) regression employing Stepwise Backward Elimination, and subsequent assumption tests including Jarque-Bera, Breusch-Pagan, Moran's I, and multicollinearity checks. Upon identifying spatial autocorrelation and heteroscedasticity, Geographically Weighted Regression (GWR) was applied to address spatial heterogeneity. Ordinary Least Squares (OLS) analysis identified Change in tourist arrival ratio per population, environmental health workforce ratio per land area, and community healthcare workforce ratio per land area as associated factors with change in COVID-19 prevalence. The Geographically Weighted Regression (GWR) model, with a higher R^2^ value of 0.66, better accounted for regional variations, especially in central and eastern regions. The findings indicate that traveler mobility and the spatial distribution of community health workers are linked to increased COVID-19 prevalence, whereas environmental health workers are associated with a protective result, but these are associations at the aggregate (district/city) level and may be influenced by confounding or reverse causation. Structural factors such as unequal access to resources, healthcare, and sanitation, driven by tourism-induced social inequality, contribute to the disproportionate impact of COVID-19 on vulnerable communities, making it essential for policymakers to address these disparities to protect both local populations and visitors. The study recommends regulating risk-based tourist activities, expanding the environmental health workforce, and enhancing spatial monitoring systems to inform evidence-based health policy.

## 1 Background

The COVID-19 pandemic, formally recognized as a global health emergency by the World Health Organization (WHO) on March 12, 2020, it has significantly altered multiple facets of global society, with long-lasting implications for public health. In Indonesia, although case numbers have significantly declined since the peak, challenges in controlling transmission remain [1,2]. As of early May 2025, several neighboring countries have experienced a resurgence in COVID-19 cases. Singapore reported 14,200 active cases, Thailand recorded over 69,000 cases, and Hong Kong reported a test positivity rate of 13.66 % [3]. Central Java, one of the most densely populated provinces in Indonesia, is experiencing a relatively high prevalence of COVID-19. In 2022, the province recorded a test positivity rate of 40.9 %, substantially exceeding the World Health Organization's recommended standard of below 5 % [2,4]. This indicates continuing challenges in the province's pandemic management. This situation reflects a complex interaction of social, economic, health, and environmental determinants that disproportionately impact vulnerable communities. The rise in COVID-19 prevalence highlights systemic inequities in health access, where the marginalized suffer disproportionately, reinforcing the importance of a socially just approach to pandemic management [[5], [6], [7]].

The progression of COVID-19 in Central Java has been shaped by structural factors such as unequal access to healthcare, poverty, education, and sanitation, which exacerbate inequities within communities, making it essential to address these underlying causes to protect vulnerable populations. This study adopts an ecological framework alongside the Social Determinants of Health (SDOH) theory, which primarily links population health to social and environmental factors [8]. High population density is associated with the spread of COVID-19. Studies in Malaysia revealed a strong positive correlation between population density and COVID-19 transmission (*r* = 0.912, *p* < 0.001), indicating that higher density was associated with an increase in active cases [9]. In Indonesia, high mobility in densely populated areas has further intensified COVID-19 transmission [10].

Low education levels limit understanding of health protocols, associated with increased transmission [11]. Furthermore, socioeconomic factors such as poverty and unemployment are linked to increased vulnerability, consistent with findings that countries with lower Index of Pandemic Management (IPM) scores experience higher incidence and mortality rates [10]. Limited access to sanitation and safe drinking water further increases the risk of disease transmission [12,13]. Alterations in forest cover and sanitation infrastructure are associated with environmental determinants of health [14,15]. With respect to healthcare provision, effective pandemic response depends on the equitable allocation of healthcare personnel. Evidence from Nepal indicates that weak health systems correlate with increased fatality rates during case surges [16]. These findings reflect the need to strengthen social, environmental, and healthcare system determinants to effectively manage COVID-19 in Central Java [14].

Further, despite extensive research on the determinants of COVID-19 transmission, the majority of studies have centered on national or global analyses. Research on the association of social, economic, and environmental factors with regional conditions, accounting for local contexts, remains limited. Specifically in Central Java, Indonesia, research using spatial analysis to examine COVID-19 transmission dynamics is underexplored, mainly those addressing regional disparities in population density and land area. Therefore, this deficiency must be addressed through a rigorous, data-driven spatial approach to uncover the determinants of COVID-19 transmission at the provincial level, particularly in Central Java.

This study examines the association between changes in sociodemographic, healthcare, and environmental variables and the rise in COVID-19 prevalence, accounting for regional disparities in population size and area. The findings of this study are expected to inform a policy framework for mitigating the pandemic. By integrating these variables into a spatial analysis model, the research aims to provide new insights into the relationships among socio-economic changes, healthcare quality, and environmental conditions in relation to surges in COVID-19 prevalence.

## 2 Materials and methods

### 2.1 Study area

**Fig. 1:**
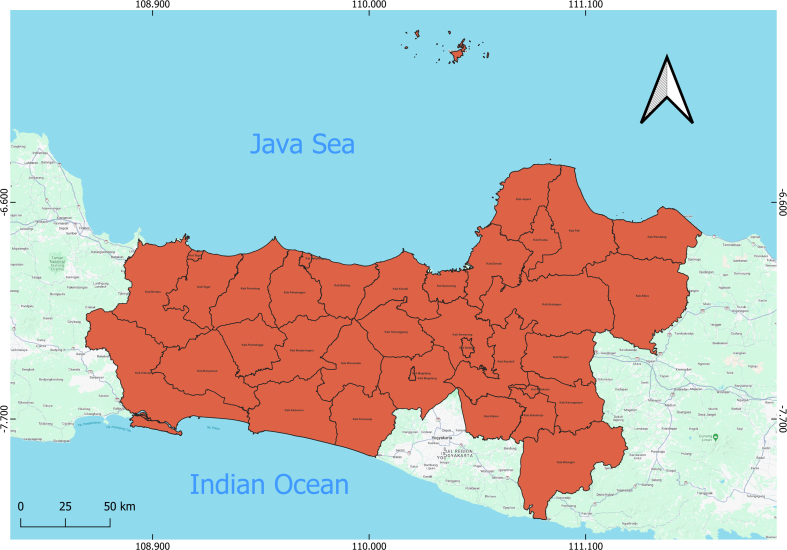
Study Location: Regencies and Cities of Central Java Province.

This study focuses on Central Java Province, located in the central part of Java Island, Indonesia, encompassing the coordinates 5°40′–8°30’ South Latitude and 108°30′–111°30′ East Longitude. It is bordered by the Java Sea to the north, the Yogyakarta Special Region and the Indian Ocean to the south, West Java Province to the west, and East Java Province to the east. Central Java Province plays a strategic role as a corridor between the western and eastern regions of Java Island. It is composed of 35 administrative divisions including 29 districts and 6 municipalities. By 2021, the province's population had reached approximately 37.5 million, making it one of the most populous in Indonesia. The region is renowned for its diverse tourist attractions, ranging from historical landmarks to natural landscapes. Tourism remains a growing and vital sector of the regional economy [17]. This region, with its diverse population and varying levels of healthcare infrastructure, offers a unique setting to explore how socio-demographic, environmental, and healthcare factors intersect to influence COVID-19 transmission patterns (See Fig. 1).

### 2.2 Data selection and pre-processing

Empirical data from the 2021–2022 period were obtained from the Central Java Provincial Health Department and the Central Java Regional Office of Statistics. To ensure accuracy and reliability in the analysis, multiple validation methods were employed. The dataset is classified as public information and is accessible through the official websites of the Central Java Provincial Statistics Agency (https://jateng.bps.go.id/id) and the Central Java Provincial Health Office, specifically, the 2021 data at (https://dinkesjatengprov.go.id/v2018/dokumen/Profil_Kesehatan_202) and the 2022 data at (https://dinkesjatengprov.go.id/v2018/dokumen/Buku_Profil_Kesehatan_2022). The data were selected based on the Social Determinants of Health (SDOH) framework, putting an emphasis on how conditions related to birth, development, residence, occupation, and aging are strongly associated with regional health outcomes. Relevant variables from prior research were also incorporated, depending on data availability [14].

**Fig. 2:**
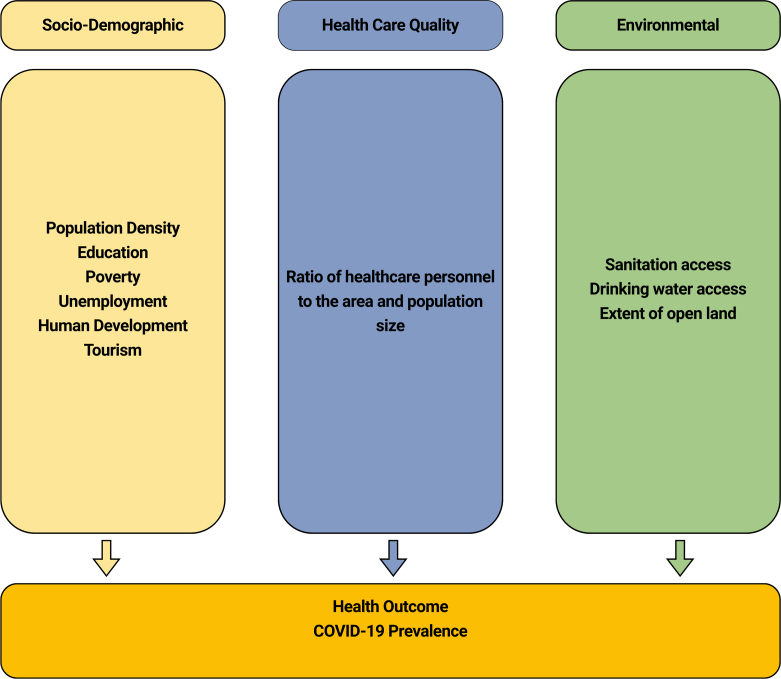
Research Framework (Modification of Social Determinants of Health (SDOH).

This analytical framework, aligned with the Social Determinants of Health (SDOH), was informed by prior empirical studies. COVID-19 incidence is influenced by the interplay of socio-demographic, environmental, and healthcare factors. Variations in population density are closely linked to mobility patterns [9,18]. Limited education intensifies existing challenges, while poverty further increases vulnerability [10,11]. Poor sanitation and environmental conditions significantly contribute to increased risk [12,13]^.^ Further, the strategic allocation of healthcare personnel is critical to effective mitigation efforts [16] (See Fig. 2).

To assess the correlation between variations in sociodemographic factors, healthcare services, and environmental conditions hypothesized to influence COVID-19 prevalence, this study examined regional disparities in population size and geographic area. This approach enhances the accuracy of findings and informs evidence-based spatial policy by calculating ratios relative to population size and geographic area [19]. The study also analyzed changes and disparities in data from 2021 to 2022 to identify trends potentially associated with rising COVID-19 prevalence [20] (See Table 1).

**Table 1:** Explanatory Variable and Preprocessing Data.

Category	Variable	Code	Explanatory Variable and Calculation Formula	Source
Dependent Variable	Increase in COVID-19 Prevalence per 100,000 Population [21]	PREV_COVID	Calculate the Prevalence per 100,000 Population for each year (the newer year and the older year). The formula to calculate the prevalence per 100,000 population is: COVID-19 Prevalence per 100,000 Population = (Number of COVID-19 Cases / Total Population) × 100,000 Calculate the Increase in Prevalence: After calculating the prevalence for both years (the newer year and the older year), the change in prevalence is determined by subtracting the prevalence of the older year from the prevalence of the newer year: Change in Prevalence = Prevalence of Newer Year - Prevalence of Older Year	Number of Covid-19 Cases: Central Java Provincial Health Office Total Population: Statistics Agency of Central Java Province
Socio-Demographic	Change in Population Density (People/100 Km^2^) [22]	KPD_PENDUK	Calculate Population Density per 100 Km^2^ for each year (the newer year and the older year). The formula to calculate population density is: Population Density per 100 Km^2^ = (Total Population / Land Area (Km^2^)) × 100 Calculate the Change in Population Density: After calculating the population density per 100 km^2^ for both years (the newer year and the older year), the change in population density is determined by subtracting the density of the older year from the density of the newer year: Change in Population Density = Density of Newer Year - Density of Older Year	Statistics Agency of Central Java Province
	Change in Ratio of Population with Poor Education per 1000 Population (>15 Years) [23]	RATIO_PENDU	Calculate the Ratio of Population with Poor Education per 1000 Population (>15 Years) for each year (the newer year and the older year). The formula for calculating the ratio of poor education is: Ratio of Population with Poor Education per 1000 Population (>15 Years) = (Number of Population (>15 Years) Who Did Not Graduate from the 9-Year Compulsory Education Program or Junior High School / Total Population (>15 Years)) × 1000 Calculate the Change in the Ratio: After calculating the ratio of population with poor education per 1000 population (>15 years) for both years (the newer year and the older year), the change in ratio can be determined by subtracting the ratio of the older year from the ratio of the newer year: Change in Ratio = Ratio of Newer Year - Ratio of Older Year	Statistics Agency of Central Java Province
	Change in Poverty Ratio per 10,000 Population [24]	RATIO_KEMIS	Calculate the Poverty Ratio per 10,000 Population for each year (the newer year and the older year). The formula to calculate the poverty ratio is: Poverty Ratio per 10,000 Population = (Number of Poor People / Total Population) × 10,000 Calculate the Change in the Ratio: After calculating the poverty ratio per 10,000 population for both years (the newer year and the older year), the change in the ratio can be determined by subtracting the ratio of the older year from the ratio of the newer year: Change in Ratio = Ratio of Newer Year - Ratio of Older Year	Statistics Agency of Central Java Province
	Change in Unemployment Ratio per 10,000 Population [25]	RATIO_PENGA	Calculate the Unemployment Ratio per 10,000 Population for each year (the newer year and the older year). The formula for calculating the unemployment ratio is: Unemployment Ratio per 10,000 Population = (Number of Unemployed People / Total Population) × 10,000 Calculate the Change in the Ratio: After calculating the unemployment ratio per 10,000 population for both years (the newer year and the older year), the change in the ratio can be determined by subtracting the ratio of the older year from the ratio of the newer year: Change in Ratio = Ratio of Newer Year - Ratio of Older Year	Statistics Agency of Central Java Province
	Change in Human Development Index (HDI) (%) [11]	IPM_PERUB	Calculate the Change in HDI: Subtract the HDI of the previous year from the HDI of the most recent year. Change in HDI = HDI of Newer Year - HDI of Previous Yea	Statistics Agency of Central Java Province
	Change in Tourist Arrival Ratio per 1000 Population [26]	RATIO_TURIS	Calculate the Tourist Arrival Ratio per 1000 Population for each year (the newer year and the older year). The formula for calculating the tourist arrival ratio is: Tourist Arrival Ratio per 1000 Population = (Number of Local Tourists + Number of International Tourists) / Total Population × 1000 Calculate the Change in the Ratio: After calculating the tourist arrival ratio per 1000 population for both years (the newer year and the older year), the change in the ratio can be determined by subtracting the ratio of the older year from the ratio of the newer year: Change in Ratio = Ratio of Newer Year - Ratio of Older Year	Statistics Agency of Central Java Province
Healthcare	Change in Healthcare Workforce Ratio per 1000 Km^2^ of Land Area [27]	RATIO_TKESW	Calculate the Healthcare Workforce Ratio per 1000 Km^2^ for each year (the newer year and the older year). The formula for calculating the healthcare workforce ratio is: Healthcare Workforce Ratio per 1000 Km^2^ = (Total Number of Healthcare Workers / Land Area (Km^2^)) × 1000 Calculate the Change in the Ratio: After calculating the healthcare workforce ratio per 1000 Km^2^ for both years (the newer year and the older year), the change in ratio can be determined by subtracting the ratio of the older year from the ratio of the newer year: Change in Ratio = Ratio of Newer Year - Ratio of Older Year	Total Number of Healthcare Workers: Health Department of Central Java Province Land Area: Statistics Agency of Central Java Province
	Change in Healthcare Workforce Ratio per 1 million Population [28]	RATIO_TKESP	Calculate the Healthcare Workforce Ratio per 1 million Population for each year (the newer year and the older year). The formula for calculating the healthcare workforce ratio is: Healthcare Workforce Ratio per 1 Million Population = (Total Number of Healthcare Workers / Total Population) × 1000,000 Calculate the Change in the Ratio: After calculating the healthcare workforce ratio per 1 million population for both years (the newer year and the older year), the change in ratio can be determined by subtracting the ratio of the older year from the ratio of the newer year: Change in Ratio = Ratio of Newer Year - Ratio of Older Year	Total Number of Healthcare Workers: Health Department of Central Java Province Total Population: Statistics Agency of Central Java Province
	Change in Community Healthcare Workforce Ratio per 1000 Km^2^ of Land Area [29]	RATIO_TKOMW	Calculate the Community Healthcare Workforce Ratio per 1000 Km^2^ for each year (the newer year and the older year). The formula to calculate the community healthcare workforce ratio is: Community Healthcare Workforce Ratio per 1000 Km^2^ = (Number of Public Health Workers + Number of Environmental Health Workers + Number of Nutritionists) / Land Area (Km^2^) × 1000 Calculate the Change in the Ratio: After calculating the community healthcare workforce ratio per 1000 Km^2^ for both years (the newer year and the older year), the change in ratio can be determined by subtracting the ratio of the older year from the ratio of the newer year: Change in Ratio = Ratio of Newer Year - Ratio of Older Year	Number of Public Health Workers, Number of Environmental Workers, Number of Nutritionists: Health Department of Central Java Province Land Area: Statistics Agency of Central Java Province
	Change in Community Healthcare Workforce Ratio per 1 million Population [30]	RATIO_TKOMP	Calculate the Community Healthcare Workforce Ratio per 1 million Population for each year (the newer year and the older year). The formula for calculating the community healthcare workforce ratio is: Community Healthcare Workforce Ratio per 1 Million Population = (Number of Public Health Workers + Number of Environmental Health Workers + Number of Nutritionists) / Total Population × 1000,000 Calculate the Change in the Ratio: After calculating the community healthcare workforce ratio per 1 million population for both years (the newer year and the older year), the change in ratio can be determined by subtracting the ratio of the older year from the ratio of the newer year: Change in Ratio = Ratio of Newer Year - Ratio of Older Year	Number of Public Health Workers, Number of Environmental Workers, Number of Nutritionists: Health Department of Central Java Province Total Population: Statistics Agency of Central Java Province
	Change in Environmental Health Workforce Ratio per 1000 Km^2^ of Land Area [31]	RATIO_TKLIW	Calculate the Environmental Health Workforce Ratio per 1000 Km^2^ for each year (the newer year and the older year). The formula for calculating the environmental health workforce ratio is: Environmental Health Workforce Ratio per 1000 Km^2^ = (Number of Environmental Health Workers / Land Area (Km^2^)) × 1000 Calculate the Change in the Ratio: After calculating the environmental health workforce ratio per 1000 Km^2^ for both years (the newer year and the older year), the change in ratio can be determined by subtracting the ratio of the older year from the ratio of the newer year: Change in Ratio = Ratio of Newer Year - Ratio of Older Year	Number of Environmental Workers: Health Department of Central Java Province Land Area: Statistics Agency of Central Java Province
	Change in Environmental Health Workforce Ratio per 1 million Population [31]	RATIO_TKLIP	Calculate the Environmental Health Workforce Ratio per 1 Million Population for each year (the newer year and the older year). The formula for calculating the environmental health workforce ratio is: Environmental Health Workforce Ratio per 1 Million Population = (Number of Environmental Health Workers / Total Population) × 1000,000 Calculate the Change in the Ratio: After calculating the environmental health workforce ratio per 1 million population for both years (the newer year and the older year), the change in the ratio can be determined by subtracting the ratio of the older year from the ratio of the newer year: Change in Ratio = Ratio of Newer Year - Ratio of Older Year	Number of Environmental Workers: Health Department of Central Java Province Total Population: Statistics Agency of Central Java Province
Environmental	Change in Household Access to Adequate Sanitation Ratio per 1000 Households [32]	RATIO_SAN	Calculate the Household Access to Adequate Sanitation Ratio per 1000 Households for each year (the newer year and the older year). The formula for calculating this ratio is: Household Access to Adequate Sanitation Ratio per 1000 Households = (Number of Households with Access to Adequate Sanitation / Total Number of Households) × 1000 Calculate the Change in the Ratio: After calculating the household access to adequate sanitation ratio per 1000 households for both years (the newer year and the older year), the change in ratio can be determined by subtracting the ratio of the older year from the ratio of the newer year: Change in Ratio = Ratio of Newer Year - Ratio of Older Year	Household Access to Adequate Sanitation: Health Department of Central Java Province Total Number of Households: Statistics Agency of Central Java Province
	Change in Households with Access to Safe Drinking Water Ratio per 1000 Households [33]	RATIO_AIR	Calculate the Household Access to Safe Drinking Water Ratio per 1000 Households for each year (the newer year and the older year). The formula for calculating this ratio is: Household Access to Safe Drinking Water Ratio per 1000 Households = (Number of Households with Access to Safe Drinking Water / Total Number of Households) × 1000 Calculate the Change in the Ratio: After calculating the household access to safe drinking water ratio per 1000 households for both years (the newer year and the older year), the change in ratio can be determined by subtracting the ratio of the older year from the ratio of the newer year: Change in Ratio = Ratio of Newer Year - Ratio of Older Year	Household with Access to Safe Drinking Water: Health Department of Central Java Province Total Number of Households: Statistics Agency of Central Java Province
	Change in Forest Area Ratio per 1000 Km^2^ of Land Area [34]	RATIO_HUTAN	Calculate the Forest Area Ratio per 1000 Km^2^ for each year (the newer year and the older year). The formula to calculate the forest area ratio is: Forest Area Ratio per 1000 Km^2^ = (Forest Area / Land Area (Km^2^)) × 1000 Calculate the Change in the Ratio: After calculating the forest area ratio per 1000 Km^2^ for both years (the newer year and the older year), the change in ratio can be determined by subtracting the ratio of the older year from the ratio of the newer year: Change in Ratio = Ratio of Newer Year - Ratio of Older Year	Forest Area: Health Department of Central Java Province Total Number of Households: Statistics Agency of Central Java Province

### 2.3 Variable preliminary refutation

Before engaging in multivariate modeling utilizing a spatial regression analysis, an initial examination is conducted through the implementation of a Pearson correlation test. This test is instrumental in identifying variables that exhibit a certain degree of association with the outcome, which is particularly advantageous in contexts characterized by high-dimensional data where the quantity of potential predictors is substantial. The threshold of 0.25 functions as a preliminary screening criterion. This permits the retention of those variables that may exhibit weak or moderate correlations, yet hold potential relevance when integrated with other variables within a multivariate framework. Furthermore, this approach can facilitate model simplification and enhance interpretability [35]. The 0.25 threshold for *p*-values is frequently designated as the “entry feasible threshold” for predictors during the bivariate phase preceding multivariate selection. It is important to note that the 0.25 threshold on the Pearson correlation represents merely an initial selection phase, rather than a conclusive determination of significance [36].

### 2.4 Spatial autocorrelation

To examine spatial patterns of COVID-19 prevalence, Moran's I statistic was used to assess global spatial autocorrelation. This analysis provided insight into the spatial distribution of rising COVID-19 cases and related variables, and enabled the identification of significant clusters and patterns [37]. Additionally, Local Indicators of Spatial Association (LISA) maps were constructed to highlight the contribution of individual regions to the overall spatial structure. LISA clusters are classified into four types: High-High (HH), Low-Low (LL), Low-High (LH), and High-Low (HL). The HH and LL clusters indicate significant spatial groupings where a region is surrounded by neighbors with similarly high or low values, respectively [38].

### 2.5 Spatial regression modeling

Spatial regression modeling provides a methodological framework for analyzing the determinants of the observed increase in COVID-19 incidence, incorporating both spatial and non-spatial variables. The models assessed include Ordinary Least Squares (OLS), the Spatial Autoregressive Model (SAR), and the Spatial Error Model (SEM), depending on the presence of spatial dependence in the dataset [39]. The distinction between non-spatial and spatial models is based on the recognition of spatial dependence, the Spatial Autoregressive Model (SAR) accounts for spatial dependence in the dependent variable, while the SEM addresses spatial dependence in the model's error term. In addition, spatial heterogeneity is considered if it is present [40,41]. Geographically Weighted Regression (GWR) is utilized as the spatial modeling approach [42,43].

### 2.6 Validation

Several validation methods were applied to ensure the accuracy and reliability of the data used in the analysis. Pearson's correlation coefficient was first calculated to identify significant correlations among variables. Subsequently, the Variance Inflation Factor (VIF) was used to detect multicollinearity among the independent variables. In spatial analysis, Moran's I and Local Indicators of Spatial Association (LISA) are used to detect global and local spatial autocorrelations. To validate the robustness of spatial modeling techniques such as the Spatial Autoregressive (SAR) and Spatial Error Model (SEM), Lagrange Multiplier (LM) diagnostic tests and robustness checks were performed to identify the most appropriate models. When heteroscedasticity is present, Geographically Weighted Regression (GWR) is considered the preferred alternative [44]

### 2.7 Spatial regression modeling applied in this study

Spatial regression modeling in this study applied Geographically Weighted Regression (GWR) to examine factors influencing the rise in COVID-19 prevalence in Central Java. The analysis followed Moran's I test, which revealed positive spatial autocorrelation, indicating interregional dependence. Ordinary Least Squares (OLS) was initially used as a global model to assess the relationship between the dependent variable (PREV_COVID) and the independent variables. Stepwise Backward Elimination was employed for variable selection, yielding several significant predictors, as detailed in the results section [45]. The heterogeneity test showed positive results; therefore, Geographically Weighted Regression (GWR) modeling was applied to capture spatial heterogeneity through coefficients that vary across locations [42].

### 2.8 Tools and software

Microsoft Excel is used as an initial tool for data processing, including ratio and variable computations. QGIS is employed to construct spatial attributes and prepare spatial data in shapefile or spatial database formats for further analysis. GeoDA is used for spatial data analysis, including calculations of spatial autocorrelation, Moran's I statistics, and classical Ordinary Least Squares (OLS) regression, as well as spatial regression techniques such as the Spatial Autoregressive Model (SAR) and the Spatial Error Model (SEM). GWR4 is specifically used for Geographically Weighted Regression (GWR) modeling. Together, these software tools provide an integrated framework for analyzing spatial dependencies, performing regression analyses, and maintaining data integrity throughout the research process. By following this methodological approach, the study aims to ensure the validity and reliability of the models and data, thereby supporting a comprehensive understanding of the research subject.

### 2.9 Research ethics

**Fig. 3:**
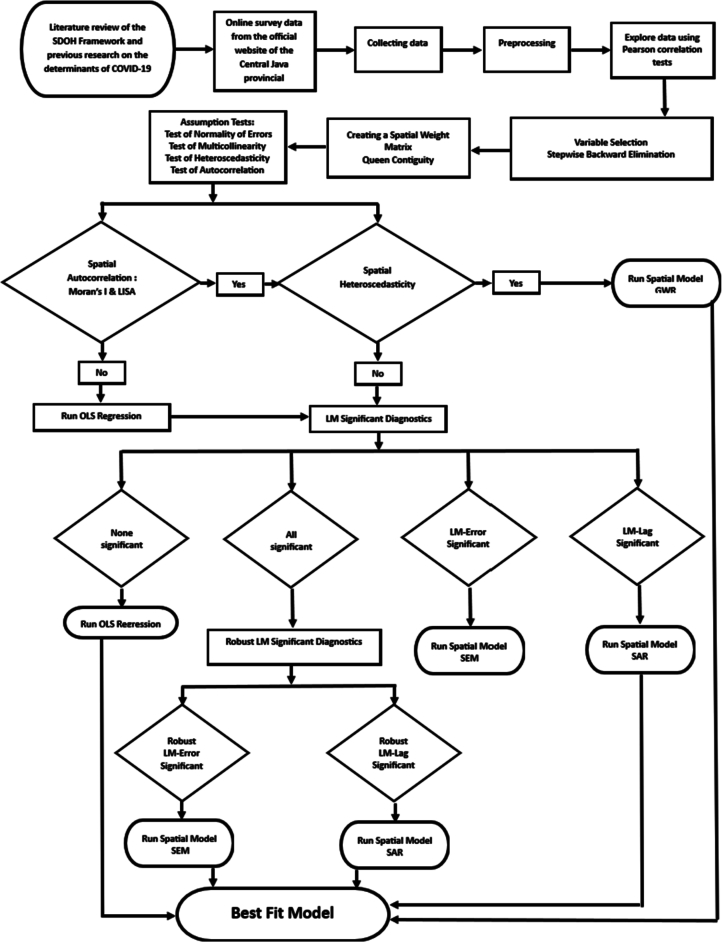
Research Flow Chart.

This study was conducted following ethical approval from the Health Polytechnic Ethics Committee of the Ministry of Semarang (Ethical Clearance Letter No. 0631/EA/KEPK/2024). To maintain research ethics, the data used were obtained from publicly accessible sources and do not violate copyright, privacy, or any relevant legal regulations. To maintain data integrity, no manipulation or modification was performed that could distort the interpretation of the findings. Transparency is maintained by thoroughly documenting data sources and properly crediting the original data owners, ensuring accountability and supporting the scientific validity of the results. For the flow of this research, see Fig. 3.

## 3 Results

Data analysis followed a systematic procedure to identify the most effective model for explaining variables associated with the rise in COVID-19 prevalence in Central Java Province. The analysis began with the identification of relevant independent variables, informed by the Social Determinants of Health (SDOH) framework and prior scholarly research, as shown in Table 2. After compiling the variable data, preprocessing was conducted, followed by bivariate analysis using Pearson's correlation coefficient.

**Table 2:** Frequency Distribution and Pearson Correlation Test Results.

Variable	Mean	Median	SD	Min	Max	Correlation (r)	P-Value
PREV_COVID	824.71	544.29	679.70	4.83	2956.68	–	–
KPD_PENDUK	1133.31	921.89	1102.71	106.12	6443.19	0.11	0.52
RATIO_PENDU	−20.91	−26.70	20.48	−68.20	24.70	0.15	0.40
RATIO_KEMIS	−79.69	−80.69	31.53	−146.07	−15.38	0.37	0.03*
RATIO_PENGA	−52.86	−21.00	79.45	−202.00	86.00	−0.40	0.02*
IPM_PERUB	0.66	0.63	0.14	0.39	0.96	−0.29	0.09*
RATIO_TURIS	817.12	501.73	826.44	−73.27	3208.96	0.20	0.24*
RATIO_TKESW	9241.02	496.03	27,809.41	189.04	133,882.71	0.41	0.01*
RATIO_TKESP	1581.09	516.80	3240.93	193.27	14,576.15	0.35	0.04*
RATIO_TKOMW	134.36	58.49	242.73	5.88	1348.46	0.61	0.00*
RATIO_TKOMP	53.46	48.86	38.96	4.21	197.49	0.33	0.05*
RATIO_TKLIW	32.73	6.94	81.63	−7.84	323.28	0.21	0.22*
RATIO_TKLIP	8.16	6.04	12.02	−7.66	49.18	0.05	0.77
RATIO_SAN	9.45	8.10	31.60	−78.00	109.10	−0.19	0.29
RATIO_AIR	−3.11	−2.90	29.17	−53.60	66.00	−0.03	0.85
RATIO_HUTAN	−795.74	−20.60	2155.57	−10,883.62	84.89	−0.08	0.63

### 3.1 Pearson correlation test

The initial step involves evaluating the linear relationship between PREV_COVID and each independent variable using Pearson's correlation test. This statistical test produces a correlation coefficient indicating the strength of the association between the two variables, along with a *p*-value that reflects the statistical significance of the correlation. Based on the correlation results, variables with a p-value less than 0.25 were considered suitable for inclusion in the Ordinary Least Squares (OLS) regression model. The 0.25 threshold was set to ensure that only variables with sufficiently meaningful correlations with the dependent variable were included in the model [46]. The adoption of a p-value threshold of 0.25 may appear atypical in comparison to the widely accepted 0.05 threshold utilized for determining statistical significance. It is imperative to emphasize that the primary objective at this juncture is not to ascertain definitive statistical significance during the preliminary correlation assessment, but rather to identify variables that exhibit potential associations. The 0.25 threshold serves as a preliminary filtering mechanism. This permits the inclusion of variables that may display weak or moderate correlations, yet hold potential relevance when integrated with other variables within the multivariate framework, thereby mitigating the risk of dismissing variables that are indeed significant if a more stringent threshold, such as 0.05, were employed at this phase. The 0.25 threshold in relation to Pearson's correlation represents merely an initial selection criterion, rather than a conclusive determination of significance [36]. It is crucial to acknowledge that this investigation scrutinizes linear associations and does not suggest causation. Employing a *p*-value threshold of 0.25 may facilitate the model's simplification and enhance its interpretability [35].

The correlation analysis (See Table 2) identified the following variables as relevant for inclusion in the Ordinary Least Squares (OLS) model: RATIO_KEMIS (Change in Poverty Ratio per 10,000 Population), RATIO_PENGA (Change in Unemployment Ratio per 10,000 Population), IPM_PERUB (Change in Human Development Index (HDI) (%)), RATIO_TOURIST (Change in Tourist Arrival Ratio per 1000 Population), RATIO_TKESW (Change in Health Workforce Ratio per 1000 km^2^), RATIO_TKESP (Change in Healthcare Workforce Ratio per 1 Million Population), RATIO_TKOMW (Change in Community Healthcare Workforce Ratio per 1000 km^2^ of Land Area), RATIO_TKOMP (Change in Community Healthcare Workforce Ratio per 1 Million Population), and RATIO_TKLIW (Change in Environmental Health Workforce Ratio per 1000 km^2^ of Land Area).

### 3.2 Stepwise backward elimination

The subsequent phase involves constructing the Ordinary Least Squares (OLS) model using the Stepwise Backward Elimination method. This process identifies relationships between the dependent variable, PREV_COVID, and nine selected independent variables. Preliminary Ordinary Least Squares (OLS) analyses revealed that some independent variables had *p*-values greater than 0.05, indicating a lack of statistical significance at the 5 % level. Consequently, a systematic reduction of the insignificant variables was carried out [45] (See Table 3).

**Table 3:** Summary of Stepwise Backward Elimination.

Var Selection	Variable	R^2^	Change R^2^	Coefficient	Change in Coefficient	highest p-value
1	All variables (9)	0.70	–	const: 947.66, RATIO_KEMIS: 2.97, RATIO_PENGA: 0.14	–	RATIO_PENGA: 0.927
2	Exclude RATIO_PENGA	0.70	0.00	const: 946.40, RATIO_KEMIS: 2.98, IPM_PERUB: −425.17	−0.14 (RATIO_PENGA)	RATIO_KEMIS: 0.292
3	Exclude RATIO_KEMIS	0.69	−0.01	const: 651.29, IPM_PERUB: −390.73, RATIO_TURIS: 0.20	−295.37 (RATIO_KEMIS), −34.61 (RATIO_PENGA)	IPM_PERUB: 0.504
4	Exclude IPM_PERUB	0.69	−0.01	const: 394.10, RATIO_TURIS: 0.21, RATIO_TKESW: −0.0003	−53.20 (IPM_PERUB)	RATIO_TKESW: 0.787
5	Exclude RATIO_TKESW	0.69	0.00	const: 394.10, RATIO_TURIS: 0.21, RATIO_TKESP: 0.10, ...	−0.0003 (RATIO_TKESW)	RATIO_TKESP: 0.568
6	Exclude RATIO_TKOMP	0.64	−0.05	const: 408.58, RATIO_TURIS: 0.24, RATIO_TKOMW: 3.60, ...	+0.06 (RATIO_TKOMP), −53.19 (RATIO_TKESP)	RATIO_TKOMP: 0.757

The process of variable selection begins with the exclusion of the variable with the highest p-value, namely RATIO_PENGA (Change in Unemployment Ratio per 10,000 Population), which has a *p*-value of 0.927. Including this variable does not result in a substantial change in the R^2^ statistic, which remains approximately 0.703, nor does it have a significant association with the model coefficients, indicating that it does not meaningfully contribute to the model. Additionally, the variable RATIO_KEMIS (Change in Poverty Ratio per 10,000 Population), with a *p*-value of 0.292, was also excluded. The R^2^ value remained at 0.690, demonstrating that the model maintains its robustness without this variable.

The subsequent phase involves the removal of the IPM_PERUB variable (Change in Human Development Index (HDI) (%)), which has a p-value of 0.504. This leads to a slight decline in R^2^ to 0.685; however, the F-statistic increases, confirming the model's continued significance. Lastly, the variable RATIO_TKESW (Change in Healthcare Workforce Ratio per 1000 km^2^ of Land Area), with a p-value of 0.787, was eliminated without any effect on the R^2^ value, which remained at 0.685, while the Adjusted R-squared showed a slight increase to 0.630. These findings suggest that excluding statistically insignificant variables can improve model efficiency without compromising its accuracy or stability.

### 3.3 Model Ordinary Least Squares (OLS)

Ultimately, following a comprehensive evaluation of various stages of variable elimination, the optimal Ordinary Least Squares (OLS) model is identified as one that includes only three independent variables: RATIO_TOURIST (Change in Tourist Arrival Ratio per 1000 Population), RATIO_TKOMW (Change in Community Healthcare Workforce Ratio per 1000 km^2^ of Land Area), and RATIO_TKLIW (Change in Environmental Health Workforce Ratio per 1000 km^2^ of Land Area). This model yields an R^2^ value of 0.64, indicating that approximately 64 % of the variation in COVID-19 prevalence is explained by these three variables. Furthermore, the model demonstrates a highly significant F-statistic (18.33) and a notably small *p*-value, suggesting that it effectively captures the associations between COVID-19 prevalence and the examined factors (See Table 4).

**Table 4:** Ordinary Least Squares (OLS) Model.

Variable	Coefficient (B)	SE	t-Statistic	P-Value	VIF
const	408.58	110.73	3.69	0.01	–
RATIO_TURIS	0.24	0.11	2.27	0.03	1.07
RATIO_TKOMW	3.6	0.51	7.08	0.01	1.25
RATIO_TKLIW	−8.07	1.68	−4.8	0.01	1.28
R^2^	0.64				
F-stat	18.33				

Low Variance Inflation Factor (VIF) values below 10 indicate minimal multicollinearity and confirm the absence of significant multicollinearity among the independent variables in the analytical framework. In applying the Ordinary Least Squares (OLS) method, a series of critical assumption tests was conducted to ensure the model's validity, including evaluations of multicollinearity, residual normality, heteroscedasticity, and spatial autocorrelation (See Table 4.

The findings from the multicollinearity assessment indicate a Condition Number of 4.78, suggesting no substantial multicollinearity among the independent variables. The residual normality test using the Jarque-Bera statistic yielded a p-value of 0.48874, confirming that the residuals are normally distributed. However, the Breusch-Pagan test revealed significant heteroskedasticity (*p* = 0.01482), which may reduce the efficiency of the coefficient estimates. Moran's I test for spatial autocorrelation in the residuals showed a slight positive autocorrelation; however, this result was not statistically significant (*p* = 0.06392). In summary, the model requires improvement, particularly in addressing heteroskedasticity and spatial dependence, which may be mitigated through the application of additional spatial modeling techniques (See Table 5).

**Table 5:** Assumption Test Results.

Test	Statistic	Value	P-Value	Notes
Multicollinearity	Condition Number	4.78	–	No significant multicollinearity
Residual Normality	Jarque-Bera Test	1.43	0.49	Residuals are normally distributed
Heteroskedasticity	Breusch-Pagan Test	10.49	0.01	There is a heteroskedasticity problem
Spatial Autocorrelation	Moran's I (error)	0.18	0.06	Positive spatial autocorrelation observed

### 3.4 Spatial autocorrelation test

Spatial autocorrelation measures the degree of association between variable values across different geographic locations. The analysis was conducted using GeoDA version 1.22, with the Spatial Weights Matrix constructed through the Queen Contiguity approach. Moran's I statistic and Local Indicators of Spatial Association (LISA) were then applied to evaluate spatial dependence. This matrix defines the spatial relationships among neighboring regions, which is essential for identifying the spatial patterns inherent in the dataset [43] (See Table 6).

**Table 6:** Spatial Weights Matrix.

Mean neighbors	median neighbors	Min- Max neighbors	% non-zero
4.23	4	1–8	12.08 %

Once the proximity matrix has been established, the next phase involves performing the spatial autocorrelation test. This test identifies and quantifies spatial interdependence among variable values across different geographic locations. In the present study, spatial autocorrelation was assessed using Moran's I, a widely recognized measure for evaluating the extent to which observed variables exhibit spatially structured patterns [38].

**Fig. 4:**
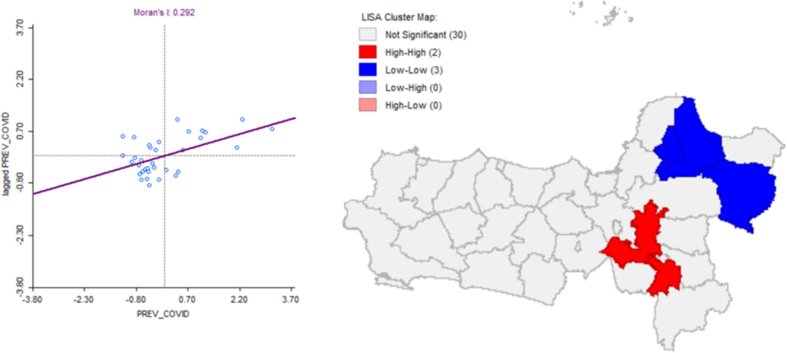
Moran's I Prev_Covid Scatter Plot with Local Indicators of Spatial Association (LISA) Cluster Map Prev_Covid.

The Moran's I statistic of 0.292 indicates a low positive spatial autocorrelation, suggesting that areas with high PREV_COVID values tend to be located near other areas with similarly high values. The scatterplot of Moran's I demonstrates a positive relationship between lagged and current PREV_COVID values, despite some dispersion among the data points. This analysis was further refined using Local Indicators of Spatial Association (LISA) mapping, which identified locally significant spatial clusters, such as High-High and Low-Low, providing a more detailed understanding of COVID-19 clustering patterns. These findings confirm the presence of spatial associations that should be considered in regional epidemiological assessments (See Fig. 4).

### 3.5 Model spatial geographically weighted regression (GWR)

This study employed Geographically Weighted Regression (GWR) to examine spatial autocorrelation and heteroscedasticity in the data related to the spread of COVID-19. Using PREV_COVID as the dependent variable and RATIO_TURIS, RATIO_TKOMW, and RATIO_TKLIW as independent variables, Gaussian-type Geographically Weighted Regression (GWR) models with optimal bandwidths were identified using the Golden Section Search method based on Cross-Validation criteria. This approach allows for localized adjustments to regression coefficients, providing a more detailed spatial understanding of the relationships between variables across different geographic locations [47].

The application of the Golden Section Search method for bandwidth selection resulted in an optimal bandwidth of 155.67, with a corresponding Cross-Validation (CV) value of 240,366.273, indicating the best model performance within the Geographically Weighted Regression (GWR) framework. Accurate bandwidth selection is essential for effectively capturing local spatial variability (See Table 7). Comparative analyses between Ordinary Least Squares (OLS) and Geographically Weighted Regression (GWR) models show that, while Ordinary Least Squares (OLS) provides global estimates, Geographically Weighted Regression (GWR) more effectively captures local heterogeneity, resulting in higher R^2^ values and lower CV values. This localized approach improves the precision of spatially varying relationships among independent variables across regions, thereby offering a stronger foundation for policy formulation informed by specific spatial characteristics [48].

**Table 7:** Bandwidth Selection.

Bandwidth Selection Method	Bandwidth Value	Criterion Value (CV)
Golden Section Search	155.67	240,366.273

The comparative analysis presented in the table highlights the significant advantages of the Geographically Weighted Regression (GWR) model in explaining the variance in COVID-19 prevalence increases, compared to the Ordinary Least Squares (OLS) model. The Ordinary Least Squares (OLS) model has an intercept coefficient of 408.58, with a standard error of 110.73 and a t-statistic of 3.69. In contrast, the Geographically Weighted Regression (GWR) model, while showing a slightly lower intercept of 405.51, exhibits a substantially reduced standard error of 15.74 and a remarkably high t-statistic of 25.75. These results accentuate the robustness and local relevance of the Geographically Weighted Regression (GWR) coefficients across different regions (See Table 8).

**Table 8:** Comparison of Ordinary Least Squares (OLS) and Geographically Weighted (GWR) Model Results for Variable Coefficients, Standard Errors, and Statistical Significance.

Model	OLS				GWR			
Variable	Coefficient	SE	t-Statistic	Significant	Coefficient	SE	t-Statistic	Significant
Intercept	408.58	110.73	3.69	Yes	405.51	15.74	25.75	Yes
RATIO_TURIS	0.24	0.11	2.27	Yes	0.25	0.03	7.83	Yes
RATIO_TKOMW	3.60	0.51	7.08	Yes	3.62	0.07	53.74	Yes
RATIO_TKLIW	−8.07	1.68	−4.80	Yes	−8.13	0.28	−29.24	Yes
CV	241,174.80				240,041.07			
R^2^	0.64				0.66			
Adjusted R^2^	0.59				0.60			

For the RATIO_TURIS variable, both models produced positive and statistically significant coefficients, with a value of 0.24 in the Ordinary Least Squares (OLS) model and a slight increase to 0.25 in the Geographically Weighted Regression (GWR) model. The lower standard errors and higher t-statistic in the Geographically Weighted Regression (GWR) model (7.83) support the conclusion that the tourism sector is strongly associated with the spread of COVID-19, with effects varying by geographic location.The RATIO_TKOMW variable also showed highly significant positive associations, with coefficients of 3.60 in the Ordinary Least Squares (OLS) model and 3.62 in the Geographically Weighted Regression (GWR) model. The exceptionally high t-statistic in the Geographically Weighted Regression (GWR) analysis (53.74) further underscores the critical role of community health personnel in spatially influenced epidemiological dynamics. Meanwhile, the RATIO_TKLIW variable exhibited notable negative coefficients in both models (−8.07 in OLS and − 8.13 in GWR), suggesting a protective effect of environmental health practitioners in mitigating the rise in case incidence.

Overall, the results of the Geographically Weighted Regression (GWR) model indicate an improvement in the coefficient of determination (R^2^ = 0.66) compared to the Ordinary Least Squares (OLS) model (R^2^ = 0.64). Additionally, the adjusted R^2^ also shows a slight improvement (0.60 vs. 0.59), along with a lower Cross-Validation (CV) value. These findings confirm the presence of spatial heterogeneity in the factors associated with COVID-19 prevalence in Central Java. Unlike Ordinary Least Squares (OLS), which produces global coefficients, Geographically Weighted Regression (GWR) captures local variations at the district/city level, offering deeper insights into the contextual epidemiological determinants.

The advantages of Geographically Weighted Regression (GWR) in identifying spatial variations carry significant policy implications. First, the findings underline that COVID-19 control strategies should not be uniformly applied across all regions. For example, interventions targeting tourist mobility should focus on areas with high RATIO_TURIS coefficients, while efforts to strengthen community health worker capacity are more effective in regions with significant RATIO_TKOMW coefficients. The deployment of community health workers should prioritize areas with negative coefficients, indicating a greater potential for prevention. This approach is consistent with WHO recommendations. Geographically Weighted Regression (GWR) results also revealed negative coefficients for environmental health workers (RATIO_TKLIW), highlighting their protective role in reducing transmission. Therefore, increasing and strategically allocating environmental health personnel, especially in areas with wide service coverage, is essential to improve sanitation monitoring and implement environment-based prevention measures. These findings emphasize the importance of risk-based, location-specific strategies in pandemic management [49].

**Fig. 5:**
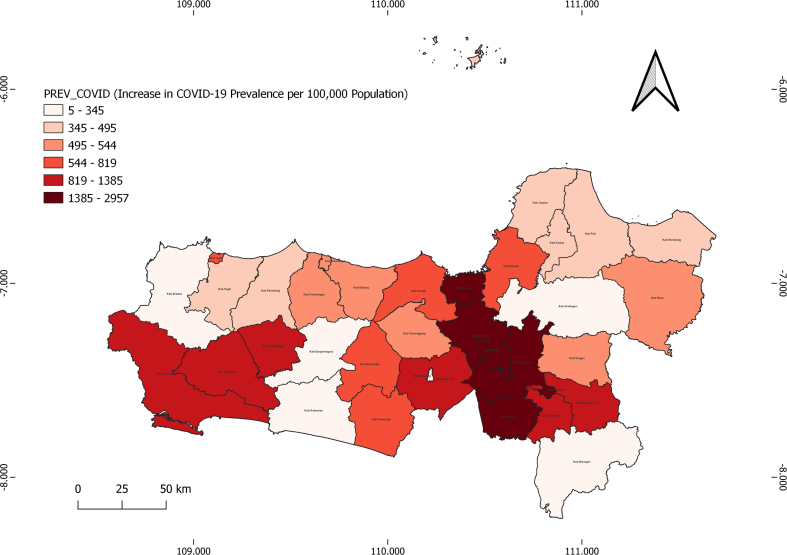
Distribution of Increase in COVID-19 Prevalence per 100,000 Population Map in Central Java.

The distribution map depicting the rise in COVID-19 incidence reveals a distinct spatial pattern, with the central to eastern regions showing the most pronounced increases. Notable areas include Semarang City, Semarang Regency, Salatiga City, Klaten Regency, and Surakarta City, with case rates reaching up to 2957 per 100,000 inhabitants. In contrast, the western and some northern regions experienced relatively modest increases. This pattern suggests the presence of spatial clusters contributing to regional disparities in the spread of COVID-19 (See Fig. 5).

**Fig. 6:**
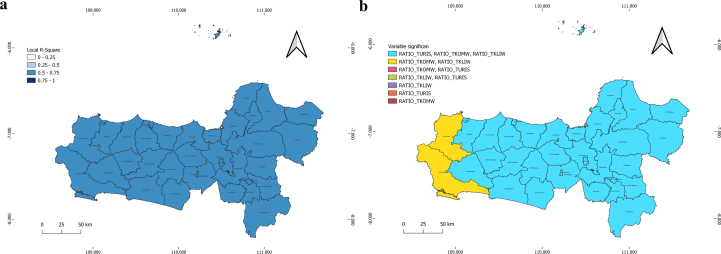
Distribution of Local R-Square Values  (a) and Variable Significance (b) Map in Central Java.

The examination of Local R-Square values supports these conclusions, showing that most regions recorded values ranging from 0.5 to above 0.75. This indicates that the Geographically Weighted Regression (GWR) model effectively captures the variability associated with the rise in COVID-19 prevalence at the local level, enabling a more nuanced and comprehensive interpretation for each region (See Fig. 6a).

A cartographic representation of key variables, namely the ratio of tourist arrivals (RATIO_TURIS), the ratio of community health workforce (RATIO_TKOMW), and the ratio of environmental health workforce (RATIO_TKIW), reveals nearly identical correlations across most administrative sectors in Brebes and Cilacap Regencies. This suggests that similar determinants contribute to the rise in COVID-19 prevalence. However, slight variations in the western regions continue to indicate the presence of spatial patterns(See Fig. 6b).

**Fig. 7:**
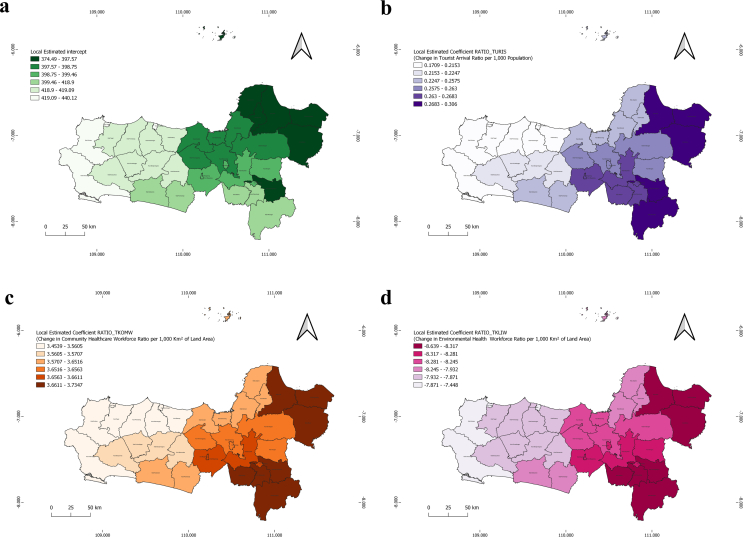
Distribution of Local Estimated Intercept (a), Ratio of Tourist Arrivals per 1000 Population (b), Community Healthcare Workforce Ratio per 1000 Km^2^ (c), Environmental Health Workforce Ratio per 1000 Km^2^ Map in Central Java (d).

In Fig. 7, variations in local intercepts across regions indicate differing baseline increases in COVID-19 prevalence independent of major predictors. Notably, the northern and eastern areas, such as Jepara Regency, Kudus Regency, Pati Regency, Rembang Regency, and Blora Regency, show elevated intercept values, suggesting the influence of additional, unmodeled factors contributing to case increases in these regions. The local coefficient for the tourist arrival ratio shows the strongest positive correlation in the eastern and southern regions, particularly in Surakarta City, Karanganyar Regency, Wonogiri Regency, Rembang Regency, and Blora Regency. This suggests that mobility linked to tourism significantly contributes to the spread of COVID-19. This finding aligns with the epidemiological principle that social mobility is a key determinant in the transmission of infectious diseases. Simultaneously, the ratio of community health workers also exhibits high coefficients in nearly the same areas. This may reflect a complex relationship between healthcare access and rising case numbers, indicating both a targeted health service response and a concentration of infection risk.

Negative coefficients observed in the ratio variables related to environmental health workers indicate a protective association, suggesting that these professionals help mitigate the spread of cases within the same geographical area. These findings highlight the critical role of environmental health practitioners in controlling both the sources and transmission of infections at the community level. Overall, the findings from the Geographically Weighted Regression (GWR) model highlight the significant spatial variations in COVID-19 prevalence across Central Java. This spatial heterogeneity suggests that a one-size-fits-all approach is insufficient to mitigate the spread of COVID-19. These insights lead to the conclusion that targeted, region-specific interventions are necessary to address local health disparities.

## 4 Discussion

This investigation evidently demonstrated that social determinants, environmental factors, and healthcare services were associated with the increased incidence of COVID-19 in Central Java Province during 2021–2022. The key finding was that the ratio of tourist arrivals (RATIO_TURIS) showed a positive correlation with COVID-19 prevalence. These results support the hypothesis that population mobility, particularly tourist influx, plays a critical role in the spread of infectious diseases. This finding aligns with international studies, which indicate that increased population mobility is closely associated with the rapid transmission of COVID-19 during the early stages of the pandemic, as the movement of travelers may accelerate virus spread due to frequent social interactions and cross-regional movement [[50], [51], [52], [53]].

In addition to tourist mobility, the proportion of community health workers (RATIO_TKOMW) showed a positive association with the incidence of COVID-19. However, these results should be interpreted with caution due to the potential for bias in determining causality. An increase in the number of community health workers in a given area is likely not a contributing factor to the rise in case numbers, but rather a response to a prior surge in infections. The expanded presence of community health professionals enables more effective screening and identification of cases, thus facilitating the detection and reporting of previously undocumented infections. This finding suggests an improvement in epidemiological surveillance quality rather than a true increase in the number of actual infections [[54], [55], [56]]. This scenario also highlights the inherent limitations of ecological study designs in determining the direction of causality [57,58].

Contrasting with community health practitioners, the proportion of environmental health professionals (RATIO_TKLIW) demonstrated a significant inverse correlation with COVID-19 prevalence. This observation suggests a potential role for the environmental health workforce in mitigating disease transmission through interventions that address the broader social determinants of health, such as sanitation, waste management, and the regulation of environmental risk factors in densely populated areas. The findings indicate that systemic health inequities, such as limited access to clean water and sanitation, may contribute to the spread of diseases like COVID-19. Studies from other countries suggest that environmentally focused strategies, which prioritize equitable access to sanitation and healthcare, may help reduce transmission rates [31,59].

The COVID-19 pandemic has highlighted significant inequities across districts and cities in Central Java, linked by various socio-economic and healthcare factors. In more urbanized areas with high tourism, such as Semarang, the prevalence of COVID-19 was notably higher. The influx of tourists and increased mobility contributed to the rapid spread of the virus, making containment efforts more difficult. Despite having a moderate healthcare workforce, the density of the population and frequent movement in these regions created significant challenges in managing the pandemic. On the other hand, rural areas like Blora experienced lower COVID-19 prevalence, likely due to less mobility and fewer tourism-related risks [52,60]. However, these regions faced their own set of challenges, particularly with limited healthcare infrastructure and a shortage of environmental health workers. The lack of resources in rural areas made it difficult to effectively control the virus, showing how disparities in healthcare access can exacerbate the effects of a pandemic, especially in less populated regions.

In contrast, districts such as Banyumas, with a combination of moderate tourism and a relatively robust healthcare workforce, were better equipped to manage the spread of COVID-19. These regions benefited from both an active healthcare presence and lower poverty levels, which helped mitigate the impacts of the pandemic [10]. Meanwhile, other areas like Tegal struggled more due to a combination of high education deficits and fewer healthcare workers. This underscores the critical role that socio-economic factors, such as healthcare access and education, play in determining how regions respond to health crises, and highlights the need for targeted interventions to address these disparities [61]. Moreover, the progression of COVID-19 in Central Java has been associated with underlying structural factors such as unequal access to healthcare, poverty, education, and sanitation. These disparities exacerbate existing health inequities within communities, disproportionately affecting vulnerable populations. Addressing these structural causes is crucial in developing long-term solutions to mitigate the effects of the pandemic and prevent future health crises. This points to the importance of a systemic, multifaceted approach to tackling COVID-19 and its social and environmental determinants [18].

These highlight the importance of addressing structural health disparities to improve overall public health outcomes. Moreover, the results from the Geographically Weighted Regression (GWR) model revealed spatial patterns in COVID-19 prevalence across regions in Central Java. However, it is important to note that these local coefficients are exploratory and should be interpreted cautiously. They highlight general trends in the data rather than definitive causal relationships, emphasizing the overall spatial patterns rather than specific causes [42]. Meanwhile, sociodemographic variables including poverty rate, unemployment rate, and Human Development Index (HDI) showed no statistically significant association in the final model. These findings stand in stark contrast to numerous international studies reporting a strong association between socioeconomic status and susceptibility to COVID-19. This discrepancy underscores the need for continued research into additional factors, including culturally influenced behaviors and community dynamics, that may contribute to vulnerability to the disease [[62], [63], [64]].

One of the primary contributions of this study was the application of Geographically Weighted Regression (GWR) to model local variations in the relationships among variables. The analysis showed that Geographically Weighted Regression (GWR) produced an R^2^ value of 0.66, slightly higher than the 0.64 obtained from the Ordinary Least Squares (OLS) model. This finding supports the conclusion that Geographically Weighted Regression (GWR) is more effective in capturing spatial heterogeneity in the spread of COVID-19 within Central Java Province. The advantages of Geographically Weighted Regression (GWR) for spatial data analysis have been well documented in the existing academic literature [65,66].

The analysis of Local Indicators of Spatial Association (LISA) produced a distribution map indicating a spatial dependence pattern, with a positive Moran's I value of 0.292; however, this association was considered weak. Areas exhibiting High-High clusters were identified in regions such as Semarang City, Semarang Regency, and Surakarta City. This suggests that COVID-19 transmission tends to concentrate in high-risk areas, reinforcing the need for region-specific interventions [67,68].

The results of this investigation have several important implications for the development of community health policy. The RATIO_TURIS variable shows the highest local coefficients in areas with significant tourism activity, including Pati Regency, Rembang Regency, Blora Regency, Surakarta City, Karanganyar Regency, and Wonogiri Regency. This underscores the need to regulate high-risk tourist mobility through measures such as capacity limits, strict health protocols, digital contact tracing at key destinations, and the establishment of maximum visitation thresholds based on local risk assessments. Previous research has shown that restricting tourist movement in high-risk areas can reduce transmission rates by up to 35 % [69].

Geographically Weighted Regression (GWR) analysis revealed a significant positive correlation with the RATIO_TKOMW variable in semi-urban districts, including Rembang Regency, Pati Regency, Blora Regency, Karanganyar Regency, Sukoharjo Regency, Klaten Regency, Wonogiri Regency, and Surakarta City. This underscores the critical role of community health workers in improving case detection in areas with limited access to secondary healthcare facilities. Policy strategies should prioritize increasing the community health worker ratio, particularly in rural areas with low reported incidence rates. Previous empirical studies have supported the claim that strengthening the community health workforce can enhance case reporting for community-based infectious diseases [70].

The RATIO_TKLIW variable exhibits markedly negative coefficients in regions such as Pati Regency, Rembang Regency, Blora Regency, Karanganyar Regency, Sukoharjo Regency, Klaten Regency, Wonogiri Regency, and Surakarta City. This trend suggests a negative association between the presence of environmental health practitioners and COVID-19 transmission, particularly in areas with wide geographical coverage. As a result, reallocating environmental health personnel to medium- to high-density areas emerges as a crucial intervention. Previous research supports these findings, showing that an equitable regional distribution of environmental health resources can effectively reduce transmission rates by improving public sanitation, monitoring environmental hazards (such as through sanitation surveillance), managing high-density areas, and overseeing waste disposal practices [31,71]. When identifying priority regions, it is strongly recommended that Pati Regency, Rembang Regency, Blora Regency, Karanganyar Regency, Wonogiri Regency, and Surakarta City be designated as central areas for intervention efforts.

Furthermore, this research underlines the critical need to establish geospatial-based health dashboards that enable real-time surveillance of disease transmission. It includes the integration of Local Indicators of Spatial Association (LISA) into pandemic preparedness frameworks to identify risk clusters based on geographic variables and resource distribution, thus supporting spatial data-informed decision-making. This initiative should be accompanied by efforts to enhance the technical capacity of governmental institutions in managing and analyzing spatial data. The World Health Organization advocates for a location-centric approach to ensure more effective interventions and accurately targeted resource allocation. Likewise, as social, environmental, and health determinants interact simultaneously, it is critical to promote cross-sectoral collaboration among health, tourism, and regional planning agencies. A multi-sectoral governance framework can significantly improve the responsiveness of pandemic management to the specific dynamics of local contexts [49,72].

While it is unequivocally true that this particular study yields significant contributions to the existing body of knowledge, it is also essential to acknowledge that it is not devoid of certain limitations that may affect the overall robustness of its findings. The analyses conducted within this research were executed utilizing secondary annual aggregate data that are collected at the district or city level, which inherently poses challenges in accurately determining the presence or absence of various measurement fallacies that could potentially skew the results. Furthermore, the ecological study design employed, which relies heavily on aggregate data, inherently constrains the researcher's ability to accurately identify causal relationships and delve into more intricate temporal dynamics, such as variations that may occur on a monthly or weekly basis. Indeed, utilizing datasets characterized by a finer temporal resolution could significantly enhance our comprehensive understanding of the dispersal patterns, providing insights that are often obscured in broader temporal analyses [73].

Important to note that the variables incorporated into the study are confined to those that are readily available within the official dataset, thereby limiting the scope of the analysis. Other critical factors that could play a vital role in influencing the outcomes, such as behavioral aspects or cultural influences specifically, community adherence to health protocols, daily mobility patterns, and the overall quality of health services, were unfortunately not included in the model. This exclusion may result in an incomplete or insufficiently nuanced explanation for certain variations observed in prevalence rates across different populations. Consequently, these limitations should be taken into account when interpreting the findings, as they could have implications for policy recommendations and future research directions. Ultimately, acknowledging these constraints allows for a more critical engagement with the study's contributions and sets the stage for further exploratory inquiries that could address these gaps in the existing literature [74].

Another limitation concerns the low spatial autocorrelation value (Moran's I), which suggests that the spatial dynamics associated with the spread of COVID-19 in Central Java are not particularly strong, or that other, more influential spatial variables may exist but were not included in the analytical framework [65]. The extrapolation of these findings requires cautious consideration, as the results are specifically applicable to the context of Central Java Province. The social, cultural, and economic characteristics unique to each region in Indonesia may significantly link to the patterns of COVID-19 transmission.

These empirical findings highlight avenues for future scholarly inquiry, particularly through longitudinal studies using more detailed spatio-temporal datasets (e.g., monthly or weekly) to gain a nuanced understanding of the dynamics underlying case proliferation. Furthermore, examining supplementary variables, such as air quality, climatic conditions, community behaviors, and socio-cultural factors, is crucial, especially in regions with unique characteristics, such as Kudus, Jepara, and Blora. Developing simulation models for spatial interventions is also critical for forecasting the impacts of region-specific policies. Integrating advanced technologies including big data analytics, machine learning, and the Internet of Things (IoT) into spatial monitoring frameworks is expected to improve the effectiveness of responses to future surges in case numbers [75].

From an academic perspective, this research contributes to the existing body of literature on factors associated with COVID-19 at the subnational level in Indonesia. During this period, most research efforts have focused primarily on national or global scales, often overlooking local disparities. The integration of Ordinary Least Squares (OLS) and Geographically Weighted Regression (GWR) in this study demonstrates how spatial analytical models can strengthen epidemiological investigations and support region-specific policy-making. This approach aligns with the evolving direction of contemporary epidemiological research, which emphasizes the importance of spatial context in understanding the spread of infectious diseases [76,77].

CONCLUSION AND RECOMMENDATIONS.

The Geographically Weighted Regression (GWR) model proved effective in this study for capturing the spatial variability associated with the escalation of COVID-19 prevalence in Central Java Province. The significant variables identified include changes in the ratio of tourists to the resident population, the density of community health workers, and the density of environmental health workers per unit area. Variations in the tourist-to-population ratio and community health workforce density were positively correlated with rising COVID-19 prevalence across most districts and cities, suggesting that increases in these variables are associated with higher reported cases. In contrast, the density of environmental health workers showed a negative correlation with COVID-19 prevalence, suggesting that increasing the number of environmental health personnel may play a critical role in mitigating the spatial spread of cases.

To effectively mitigate the escalation of COVID-19 prevalence in Central Java Province, it is imperative for the government to implement stricter management of tourist inflows and to increase the ratio of environmental health personnel based on designated areas of responsibility. These initiatives are expected to curb case growth through more targeted, spatially oriented interventions. Furthermore, to strengthen COVID-19 screening efforts, it is essential to expand the number of community health practitioners in alignment with specific service areas. Enhancing the community health workforce is anticipated to accelerate early detection and enable more rapid responses to transmission, thereby improving the overall effectiveness of epidemic control measures.

## Ethical and participation approval

All procedures conducted in this study complied with the ethical standards of the national research committee and were approved by the Health Polytechnic of Health Research Ethics Committee, Ministry of Health Semarang, under protocol number 0631/EA/KEPK/2024.

## Generative ai declaration

During the preparation of this manuscript, the author utilized ChatGPT for language processing and grammatical refinement. All content generated with the assistance of this tool was subsequently reviewed, revised, and edited by the author, who assumes full responsibility for the final version of the published article.

## Funding

This work was supported by Ministry of Health Health Polytechnic Semarang (Poltekkes Kemenkes Semarang), under grant number HK.02.03/6.1/2855/2024.

## CRediT authorship contribution statement

**Iqbal Ardiansyah:** Writing – review & editing, Validation, Supervision, Conceptualization. **Agus Subagiyo:** Data curation. **Arif widyanto:** Formal analysis. **Army Mitasari:** Writing – original draft.

## Declaration of competing interest

The authors declare that they have no known competing financial interests or personal relationships that could have appeared to influence the work reported in this paper.

## Appendix A Supplementary data

Supplementary material Image 1
