# Supplementary material for: Determinants of COVID-19 prevalence in Central Java, Indonesia: An ecological study of socio-demographic, environmental, and healthcare factors
Source: Dialogues Health. 2025 Dec 12;8:100263. doi: 10.1016/j.dialog.2025.100263 (PMC12774727; doi:10.1016/j.dialog.2025.100263)
Supplement: Supplementary file 1 — Supplementary material [file mmc1.docx]

**SUPPLEMENTAL APPENDIX**

**OLS (Ordinary Least Squares)**

Ordinary Least Squares (OLS) constitutes a traditional linear regression framework employed to quantify the relationships between independent and dependent variables through the minimization of the sum of squared discrepancies between the observed and predicted values. This framework postulates that the residuals (errors) exhibit independence and lack any spatial correlation ^1,2^.

Model:

*y_i_* = *β_0_* + *β_1_x_1i_* + *β_2_x_2i_* + ⋯ + *β_k_x_ki_* + *ϵ_i_*

Explanation:

*y_i_* : dependent variable at location *i*

*x_1i_*, *x_2i_* ,…, *x_ki_*_​_ : independent variables at location *i*

*β_0_* : intercept

*β_1_*, *β_2_* ,…, *β_k_*​ : coefficients for the independent variables

*ϵ_i_* : error term at location *i*.

**SAR (Spatial Autoregressive Model)**

The Spatial Autoregressive (SAR) model incorporates spatial dependencies present within dependent variables. This particular model encompasses the spatial lag of the dependent variable, indicating that the value of the dependent variable at a specific locale is affected by the values of the dependent variable at adjacent locales ^3^.

Model:

*y_i_* = *ρWy* + *X_i_β* + *ϵ_i_*

Explanation:

*y_i_*  : dependent variable at location *i*,

*W* : spatial weight matrix that describes spatial relationships between locations

*ρ* : autoregressive coefficient that indicates the degree of spatial dependence

*X_i_* : vector of independent variables at location *i*

*β* : vector of coefficients for the independent variables

*ϵ_i​_* : error term at location *i*

**SEM (Spatial Error Model)**

The Spatial Error Model (SEM) posits that spatial dependence is attributed to the error terms rather than the dependent variables. This particular model is applicable when the residuals derived from the Ordinary Least Squares (OLS) model exhibit signs of spatial correlations, indicating the existence of spatial associations that remain inadequately addressed within the model framework ^4^.

Model:

*y_i_* = *X_i_β* + *ϵ_i_*

Where the error term follows a spatial model:

*ϵ_i_ = λWϵ + u_i​_*

Explanation:

*y_i_* : dependent variable at location *i*

*X_i​_* : vector of independent variables at location *i*

*β* : coefficient for independent variables

*ϵ_i_* : error term at location *i*

*W* : spatial weight matrix

*λ* : coefficient indicating the influence of spatial dependence on the error term

*u_i​_* : residual error term at location *i*.

**GWR (Geographically Weighted Regression)**

Geographically Weighted Regression (GWR) permits the variation of regression coefficients contingent upon geographic context. This analytical framework is employed in instances of spatial heterogeneity, whereby the relationships between dependent and independent variables may exhibit divergence across different locales ^5^.

Model:

*y_i_ = β_0_(_ui,vi_) + β_1_(_ui,vi_)x_1i_ + β2(_ui,vi_)x_2i_* + ⋯ + *βk(_ui,vi_)x_ki_ + ϵ_i_*

Keterangan:

*y_i_* : dependent variable at location *i,*

*x_1i_, x_2i_* , … , *x_ki​_* : independent variables at location *i,*

*β_0_(_ui,vi_), β_1_(_ui,vi_)* ,…, *βk(_ui,vi_)* : coefficients varying by location (*ui,vi*)

*ϵ_i_* : error term at location *i.*

**REFERENCES**

1. Wooditch A, Johnson NJ, Solymosi R, Medina Ariza J, Langton S. Ordinary Least Squares Regression. *A Beginner’s Guide to Statistics for Criminology and Criminal Justice Using R*. Published online 2021:245-268. doi:10.1007/978-3-030-50625-4_15

2. Li PH. Linear Regression. *Numerical Methods Using Java*. Published online 2022:915-978. doi:10.1007/978-1-4842-6797-4_14

3. Kazar BM, Celik M. Theory behind the SAR Model. *SpringerBriefs in Computer Science*. 2012;0(9781461418412):7-17. doi:10.1007/978-1-4614-1842-9_2

4. Darmofal D. Spatial Lag and Spatial Error Models. *Spatial Analysis for the Social Sciences*. Published online November 5, 2015:96-118. doi:10.1017/CBO9781139051293.007

5. Wheeler DC, Páez A. Geographically Weighted Regression. *Handbook of Applied Spatial Analysis*. Published online 2010:461-486. doi:10.1007/978-3-642-03647-7_22
